# Markov Task Network: A Framework for Service Composition under Uncertainty in Cyber-Physical Systems

**DOI:** 10.3390/s16091542

**Published:** 2016-09-21

**Authors:** Abdul-Wahid Mohammed, Yang Xu, Haixiao Hu, Brighter Agyemang

**Affiliations:** 1 School of Computer Science and Engineering, University of Electronic Science and Technology of China, Chengdu 611731, China; abdulwahidmohammed@yahoo.co.uk (A.-W.M.); 201511060118@std.uestc.edu.cn (H.H.); brighteragyemang@gmail.com (B.A.); 2 School of Engineering, University for Development Studies, Tamale 00233, Northern Region, Ghana

**Keywords:** cyber-physical systems, Markov logic networks, hierarchical task networks, ontology, uncertainty reasoning

## Abstract

In novel collaborative systems, cooperative entities collaborate services to achieve local and global objectives. With the growing pervasiveness of cyber-physical systems, however, such collaboration is hampered by differences in the operations of the cyber and physical objects, and the need for the dynamic formation of collaborative functionality given high-level system goals has become practical. In this paper, we propose a cross-layer automation and management model for cyber-physical systems. This models the dynamic formation of collaborative services pursuing laid-down system goals as an ontology-oriented hierarchical task network. Ontological intelligence provides the semantic technology of this model, and through semantic reasoning, primitive tasks can be dynamically composed from high-level system goals. In dealing with uncertainty, we further propose a novel bridge between hierarchical task networks and Markov logic networks, called the Markov task network. This leverages the efficient inference algorithms of Markov logic networks to reduce both computational and inferential loads in task decomposition. From the results of our experiments, high-precision service composition under uncertainty can be achieved using this approach.

## 1. Introduction

Cyber-physical systems (CPSs) [1,2] currently offer the gateway to achieving synergy between the digital and physical worlds. With the integration of CPSs and Internet of Things (IoT) [3], the sea of interconnected devices provides an avenue for the exchange of capabilities towards the attainment of common goals. However, differences in the operations of the cyber and physical objects and the lack of adequate techniques for the dynamic formation of new functionality given high-level cross-layer services and their underlying criteria are fundamental challenges that need to be addressed. As such, a cross-layer automation and management framework, which can represent both the cyber and physical components with high fidelity, is urgently needed. It is against this background that researchers in this field have made attempts towards new abstractions and architectures that can spur on novel techniques in the development and implementation of CPSs.

Existing service-oriented architectures (SOAs) can achieve interoperable models that represent both the cyber and physical components [4,5]. As the components of CPSs increasingly grow apart, agent-based modeling techniques have been pursued to complement SOAs for autonomous discovery and management of services [6]. To achieve interoperability and scalable information management through context-awareness [7], semantic agent technology has also become apparent in the development of CPSs [8]. Semantic agents represent heterogeneous computational platforms executing complex algorithms and can encapsulate complex attributes to achieve real-time CPSs that take into account the strong interdependencies between the cyber and physical components.

Even though an agent-based workflow modeling can address inter-enterprise collaboration involving internal and ad hoc external processes [9], agent-based models for CPSs are yet to integrate a mechanism that achieves multi-threading of physical entities by providing a dynamic service composition procedure. However, the problem of composing services to achieve complex systems tasks has become practical in CPSs [10], and other important enterprise integrations, such as business-to-business processes [11,12]. Therefore, augmenting the agent-based interoperable models for CPSs with a service composition strategy that is dynamic and adaptive to the changing evolutions of systems is very crucial. Furthermore, since the dynamic evolutions of systems naturally pose partial observability problems [13,14], CPSs must be designed and operated by incorporating uncertainty into modeling for best performance in practice.

In this paper, we propose an uncertainty-based cross-layer automation and management model for CPSs. This model uses an ontological abstraction to define a hierarchical task network (HTN) [15] for dynamic service composition in CPSs. To support semantic reasoning on this model, we propose an ontology that can efficiently integrate with existing standards in sensor networks. Because a task represents any intended service with a structure in the ontology, services can be dynamically composed into desired capabilities through HTN task decomposition. Furthermore, in dealing with uncertainty, we propose a novel bridge between HTN and Markov logic networks (MLN) [16] called the Markov task network (MTN). This new framework leverages the dynamic task decomposition of HTN and the inferential power of MLN [17,18] to achieve dynamic service composition under uncertainty for CPSs. A key advantage with this approach is the reduction of both inferential and computational loads for optimal model performance. Finally, we validated the approach using a case study of automated planning in a smart home, and the results of our experiments show that high-precision service composition under uncertainty can be achieved using this approach.

## 2. Problem Description

An agent-based interoperable model for CPSs typically describes processes and interactions amongst agents between the cyber and physical components. The problem of service composition on this model can be formulated as:

$$SC_{A}=<W,T,O,R,A>$$

where:

- *W*: Denotes a *workflow*, which represents an ordering of a set of tasks to be performed.
- *T*: Denotes a *task*, which represents a sequence of operations intended for a given service.
- *O*: Denotes an *operator*, which represents resources that achieve specific tasks.
- *R*: Denotes a *role*, which represents a placeholder for a service to achieve a task.
- *A*: Denotes an *agent*, which represents complex algorithms executing on distributed environments.

Essentially, this model leverages the advantages of multi-agent coordination mechanisms, and by specifying operators and their roles, efficient actuation control in CPSs can be achieved through task assignment.

**Figure 1:**
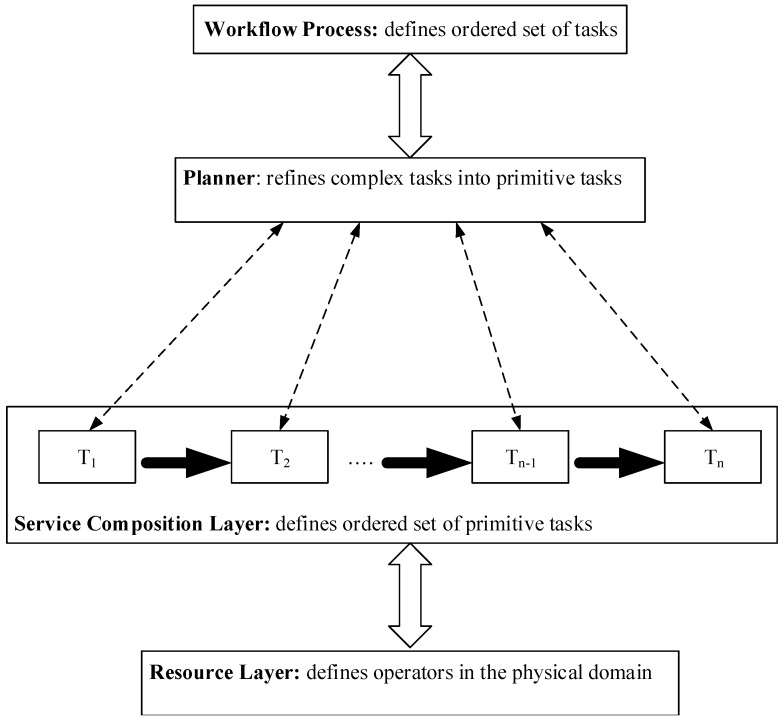
Layered view of the service composition process from a workflow process.

When the workflow is defined as a process, we achieve multi-level collaborative tasks in which each task represents a logical entity contributing to the process. As shown in Figure 1, the workflow process can be simplified by an abstraction to a service composition process of layered roles. This uses a coordinated problem-solving approach to achieve workflow management in which a planner composes primitive tasks from complex tasks in the workflow. In this way, utilities can be initiated to activate operators based on their capabilities for the execution of planned services.

Because the task ordering in the workflow generally has a hierarchical structure, a key constraint guiding the service composition process requires a planning scheme that can compose tasks into subtasks, properties and relations. From this viewpoint, the intrinsic complexity coupled with the timeliness requirement of CPSs demands a semantically-oriented approach that can specify tasks and all of their operations. However, automatically composing adaptive primitive tasks from high-level complex tasks becomes challenging when model complexity grows due to the changing evolutions of CPSs. This is also the source of uncertainty in CPSs, and good model performance can be guaranteed by pursuing novel techniques that can reduce considerably both inferential and computational loads in practice whilst incorporating uncertainty in modeling.

## 3. Hierarchical Semantic Collaborative Service Model

**Figure 2:**
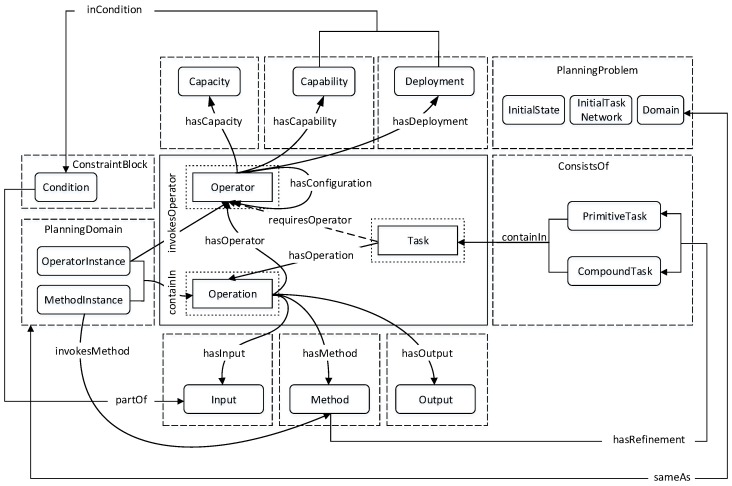
Hierarchical semantic task model.

In this section, we present a model that provides the workflow requirements of our problem description. As shown in Figure 2, this model derives its structure by extending the traditional semantics of ontology to incorporate HTN [15]. A key advantage of this design is the structural similarities between HTN and the workflow process, and the deterministic task decomposition of HTN can be leveraged for dynamic service composition. *Task* is the central logical entity of this model and represents anything that a service executes to deliver objectives, tactical results or strategy realization. Additionally, *operator* and *operation* are defined to streamline operations towards optimal performance of services. These three components, together with their extended properties, define the semantics of our hierarchical semantic task (HST) model.

### 3.1. Task

A task requires a structure that can encapsulate *resources* and some constraints. A resource can be anything abstract or concrete required to accomplish a task. Three types of resources are defined in this approach: *simple resource*; *complex resource*; and *hybrid resource*. Whilst a simple resource directly achieves a task, a complex resource depends on other resources to execute a task. A resource that shares properties of both simple and complex resources represents a hybrid resource. A typical example of a hybrid resource is a human in a fire extinguishing process. When the human is viewed as a resource in a fire tender, we can describe it as a simple resource required to move the fire tender to a fire scene. However, a human using a hand-held fire extinguisher to put out the fire becomes a complex resource, which requires a fire extinguisher as a complementing resource to perform this task.

The key property in resource modeling is *configuration*, which defines all sub-resources of a resource and provides the distinction between simple and complex resources. As shown in Listing 1, the complex resource, fire tender, consists of a fuel tank, which contains fuel as a resource for mobility, and a water tank, which also contains water as a resource for extinguishing the fire. Transporting the water to a fire scene also requires a specialized driver, which is another resource. Because some of these sub-resources can be replenished, a high frequency of replenishing resources can be avoided through *capacity* modeling. A capacity property defines the capability range of a resource.

Once resources are defined, the semantics of a task requires the assignment of resources to properties of tasks, as shown in Listing 2. *Task operation* is adopted as the sole property of every task, and this compactly represents all aspects of task planning. This representation can achieve total or partial ordering of operations and other tasks.

### 3.2. Operation

A task operation defines a *planning domain* of an HTN planning problem *P*. Specifications for both *methods* and operators of the planning domain are provided, and *P* with formal arguments *ϕ* is defined as the atomic formula ρ(P)=P(ϕ), where *ϕ* encapsulates the domain description, initial state and initial task network. For a given task instance *t* and operation *o*, the semantics of *P* is defined as $\rho \left(P\right)=\{P\left(\phi \right)⇐o\left(Operation\right)\wedge \left\{t_{1}\left(Task\right)\wedge \ldots \wedge t_{n}\left(Task\right)\right\}\}$. Clearly, from this definition, the initial state is implicitly buried in the task operation.

The representations for operator and method instances in operation are used to disambiguate between primitive and complex tasks. To specify operator and method instances, constraints and action effects are modeled as *input* and *output* requirements of operations (Listing 3 and Listing 4). Actions of operators leave effects, which can be modeled as the output property of a task operation in CPSs. For each operation, only one method instance is applicable, and the decomposition criteria are specified by a method refinement property.

### 3.3. Operator

An operator represents a resource augmented with *capability* and *deployment* properties. This augmentation encodes capacity, capability, configuration and deployment as the intrinsic properties of operators.

**Figure 3:**
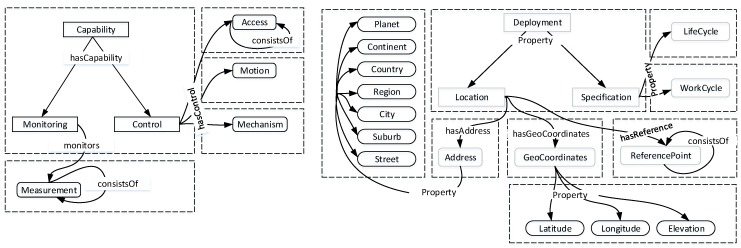
Capability and deployment modeling.

The capability property describes the ability of an operator to execute a specific set of tasks. In Figure 3, *control* and *monitoring* form the core of the capability model and simplify the capability-based search by providing enriched semantic information for efficient reasoning. With the control capability, operators can act on environments, and this is modeled in terms of properties: *access control; motion control; and mechanism control*. Whilst access control defines operations that can be performed on operators, motion control applies to all operators with a movement property. Mechanism control is also used to specify the autonomous capability of operators, such as unmanned vehicles. The monitoring capability designates the sensing properties of operators. This is liberally inclusive of anything that can monitor or convey information and can be seen from both *observation* and *signal* perspectives.

The deployment property describes the environment perspective of operators. The semantics focuses on describing an operator with respect to time, space and the projection of its status following a change in variables, such as time, or the occurrence of a predetermined event. As shown in Figure 3, complementing the deployment property are two properties: *location*; and *specification*. The location property encapsulates *physical address*, *geographic coordinates* and *reference points* to form a single descriptor denoting both absolute and relative addressing. This coordinate system uses measures of *latitude*, *longitude* and *elevation* to determine the deployments of operators. Together with physical addressing, an alternative consideration for error checking in the coordinate system is provided. In the physical address, *planet* is the top level and can be extended to include domain-specific needs. Furthermore, reference points provide relative proximities between operators in distributed deployments. This leverages the geographic coordinates to integrate different deployments. *Work cycle* and *life cycle* are two properties that define the specification property. Work cycle models the sequence of states from the start to the end of a task operation in a given deployment. The life cycle provides estimates of time required to complete a particular operation.

### 3.4. HSS Ontology

To automatically reason and interpret workflow dynamically based on the HST model, we propose a hierarchical semantic service ontology (HSS ontology). This ontology formally describes the vocabulary of the semantics of the HST model and represents an upper level conceptualization of an HTN planning problem that can integrate with existing standards in sensor networks, such as the SSN ontology [19]. The full ontology consists of 28 classes and 28 properties.

In the HSS ontology, semantic concepts describing task, operation, operator, capability, deployment, input, method and output are of the thing type. The concepts of deployment consist of location and specification. Whilst the concepts of location describe the address, geocoordinates and reference point, the concepts of specification include life cycle and work cycle. The concepts of the address begin at the level of a continent and can be extended to meet domain-specific needs. Furthermore, the capability class describes the concepts of control and monitoring. The concepts of the control class include access, mechanism and motion. The only concept under the monitoring class is measurement.

The relation between task and operation, i.e., *hasOperation*, the relation between operation and operator, i.e., *hasOperator*, the relation between operation and input, i.e., *hasInput*, the relation between operation and method, i.e., *hasMethod*, and the relation between operation and output, i.e., *hasOutput*, are modeled as object properties. Other object properties, such as *hasCapability*, which describes the relation between operator and capability, exist at the range of these relations. Data type properties are used to describe relations between address elements and range values, the relation between life cycle and range values and the relation between work cycle and range value.

**Figure 4:**
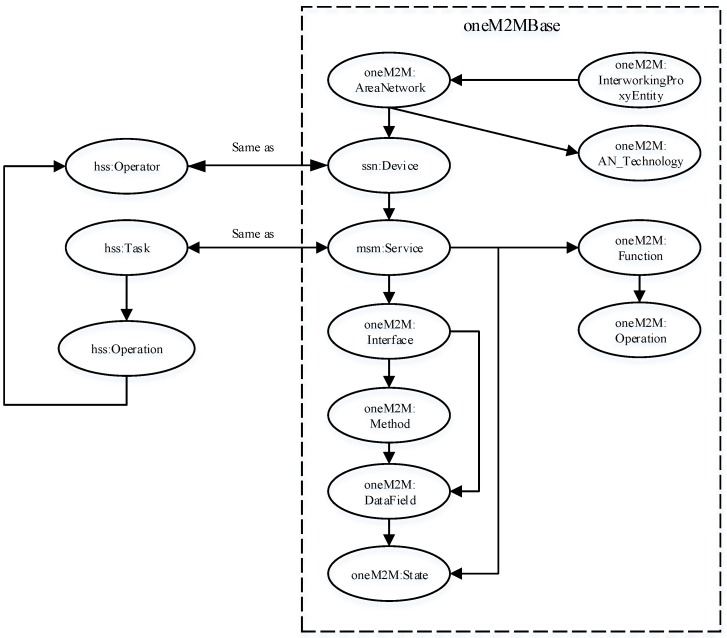
Mapping between hierarchical semantic service (HSS) ontology and oneM2Mbase ontology for generic inter-working.

A key advantage with this ontology lies in the flexibility and reusability of concepts with minimum redesign and re-development efforts. As shown in Figure 4, the oneM2M [20] base ontology proposed for generic inter-working with area networks can incorporate task processing by integrating with our HSS ontology.

As we can see, this mapping establishes equivalence between the two ontologies using a minimum set of concepts in the HSS ontology. Two concepts are deemed equivalent in two ways. First, we can establish correspondence between two concepts if they share some commonalities. Second, one concept exists in the definition of the other. For example, the correspondence between task and service is achieved because a service is required to execute a task.

## 4. Markov Task Network

We present an equivalence-preserving translation of HTN into an MLN called a Markov task network (MTN). This framework specifies a template for the ground Markov network (MN) based on our HSS ontology and leverages the inferential power of MLN to achieve non-deterministic HTN task decomposition towards dynamic service composition in CPSs. MLN models complexity using first-order logic (FOL), and models uncertainty using numerical constants attached to formulae. Following this ability of MLN to efficiently handle both complexity and uncertainty, this approach eases the difficulty of getting CPSs to function in complex and uncertain environments.

MTN can be derived from the HSS ontology by translating concepts and properties into weighted FOL formulae. This is an Interlingua-based semantics [21] involving a base language and a procedure for translating the base language into the Interlingua. Markov logic is the base language, and the translation accomplishes a combination of FOL and probabilistic graphical models to produce weighted FOL formulae according to Definition 1.

**Definition** **1.** Given a Web Ontology Language (OWL) and Markov logic (ML) as its Interlingua language, the pair $<TRANSL\left(OWL,ML\right),T_{OWL}>$ represents the Markov logic-based semantics for OWL when for every set T(OWL) of top-level forms in OWL, there exists a set T(ML) of top-level forms in Markov logic, such that: $\forall T_{1}\in T\left(OWL\right)$, $\exists T_{2}\in T\left(ML\right)$, <TRANSL(OWL,ML($T_{1},T_{2}$))>; and $\forall T_{2}\in$ T(ML), $\exists T_{1}\in$ T(OWL), <TRANSL(OWL,ML($T_{1},T_{2}$))>; where TRANSL(OWL,ML) specifies translations between top-level forms of OWL and Markov logic and $T_{OWL}$ is the set of top-level forms in Markov logic.

The OWL ontology in context denotes the HSS ontology. By specifying the semantics of OWL based on *<TRANSL(OWL,ML), $T_{OWL}$>*, the underlining theories of T(OWL) and $T\left(ML\right)\cup T_{OWL}$ are equivalent. This allows the OWL concepts to be converted into MLN using FOL, and the MLN formulae can be axiomatized similar to the OWL statements. Essentially, constraints on logical interpretations of classes and properties in OWL can also be expressed as axioms in MLN.

In the conversion process, specifications are provided for both translation TRANSL(OWL,ML) and the MLN $T_{OWL}$. First, ontological statements are translated into FOL *functions* and *predicates*. This assumes a predicate-based ontology in which classes denote unary predicates and properties represent binary predicates. In this way, all ontological statements exist as a pair <Class,Property> and can be translated as either a class or axiom of a class. Thus, an OWL class can be translated into FOL as *C(X)*, where *X* denotes all instances of *C*. As we can see in Table 1, FOL translations are provided for all crisp set properties, OWL properties and the value constraints of the property restrictions of an OWL class. For instance, an OWL property is translated into an FOL as $\forall x,yP\left(x,y\right)\Rightarrow C_{1}\left(x\right)\wedge C_{2}\left(y\right)$, where *x* and *y* respectively specify the domain and range of the property. This representation best suits an object property and can be extended to data type properties by fictionalizing range classes to represent data values.

The OWL ontology also contains additional sequence of axioms, which define logical assertions about classes, individuals and properties. In this regard, translations of some relevant axioms are also provided in Table 2. For complex formulae in rules, individual translations can be aggregated using logical connectives. The capability model is a typical case, and its subclasses can be combined into a single composite formula as ∀x,y,Access(x)∧Motion(y)⇒Control(x)∧Control(y), where x,y represent respectively individuals of the access and motion concepts of control capability.

Next, TRANSL(OWL,ML) provides a correspondence between the first-order knowledge base constructed from these FOL formulae and MLN, and it is defined as:

$$TRANSL\left(<Class,Property>,ML\right):=\left(F_{i},w_{i}\right)$$

where $F_{i}$ is a formula in FOL and $w_{i}$ denotes the weight of each formula. Thus, for any ontology-oriented HTN, we can achieve MTN based on the following proposition:

**Proposition** **1.** Given a set of ontological axioms of ontology-oriented HTN and its equivalent set of first-order weighted formulae pairs $\left(F_{i},w_{i}\right)$, then there exists a set of weighted horn logic formulae equivalent to the semantic Markov task network (MTN) of the ontology.

**Proof.**  Let *K* represent an ontology-oriented HTN and $\left(F_{i},w_{i}\right)$ denote the weighted first-order logical semantics of *K*, then Markov logic equivalence is achieved if for each formula $f_{i}$ translated from *K*; inference based on this semantics is sound and complete, and as such, adheres to Markov logic inference rules. □

When $\left(F_{i},w_{i}\right)$ is defined together with a finite set of constants, we obtain a ground MN in which each node represents a predicate appearing in the MTN, and there exists a feature for each grounding of $F_{i}$. Each weight $w_{i}$ is used to compute a log-probability, which shows the extent to which a world satisfies a formula.

The choice of weights for formulae is crucial for efficient inference in MLN since weights define the probability distribution over variables of MLN. In CPSs however, the distributed infrastructure allows the possibility of merging formulae from different distributed environments, which determines the weights of formulae. Essentially, weights of formulae can be refined by leveraging available data to automatically adjust weights through learning. Weight learning is one of the strengths of MLN and can be done either generatively or discriminatively [22].

### 4.1. Dynamic Service Composition under Uncertainty

Using HTN task decomposition, MTN can facilitate the uncertainty-based dynamic discovery and composition of capabilities of entities to achieve desired tasks. To ensure the adaptability, efficiency and flexibility of this process, this paper adopts a rule-based knowledge restructuring to provide a very tight correspondence between a subset of MN instantiated by an MLN and its relevance to reasoning requirements. As a principle and standard, all rules must be consistent with standard HTN task decomposition algorithms based on MTN.

We define a partial order planning problem as P=<s,t,O,M>, where *s* is the initial state, *t* is the partially ordered task network, *O* is a set of operator instances and *M* is a set of method instances. Because our approach holds both operator and method instances in a task operation $O_{pr}$, we can reformulate this planning problem as $P=<s,t,O_{pr}>$ where $O_{pr}$ defines the planning domain. This simplifies task decomposition based on rules, and Algorithm 1 provides a framework for formulation of rules for task decomposition on MTN.

**Algorithm 1:** Algorithm for the partial-order Markov task network (MTN). **Input:** initial state *s*, initial task network *t*, set of task operations $O_{pr}$ **Output:**
*π*, set of operator instances
 1:**function** PFD($s,t,O_{pr}$) 2:**if** w=∅ **then** 3: **return** empty plan 4:**else** 5: nondeterministically select any u∈t without predecessors in *t* 6: **if** operation of $t_{u}$ has operator instance **then** 7:  active←{(a,σ)} 8:  **if** active=∅ **then** 9:   **return** failure 10:  **end** **if** 11:  nondeterministically select any (a,σ)∈active 12:  π←PFD($\gamma \left(s,a\right),\sigma \left(t-\left\{u\right\}\right),O_{pr}$) 13:  **if** π=failure **then** 14:   **return** failure 15:  **else** 16:   **return** a.π 17:  **end** **if** 18: **else** 19:  active←{(m,σ)} 20:  **if** active=∅ **then** 21:   **return** failure 22:  **end** **if** 23:  nondeterministically select any (m,σ)∈active 24:  nondeterministically select any task network $t^{\prime }\in \delta \left(t,u,m,\sigma \right)$ 25:  **return** PFD($s,t^{\prime },O_{pr}$) 26: **end** **if** 27:**end** **if** 28:**end function**


Algorithm 1 consists of a partial order of concepts represented by a nested concept graph [23]. Concepts denote tasks, and the partial ordering of tasks depicts a method ordering based on nested ordering of subtask relations in an ontology. Thus, task decomposition methods can be invoked on the fly upon satisfying some constraints, and the nested ordering of subtasks in a decomposition hierarchy is related by $t_{1}\leq t_{2}$ to indicate that task $t_{1}$ decomposes into task $t_{2}$. By this relation, methods applicable to the decomposition of subtasks become valid only after a task is substituted by a network of its subtasks.

This algorithm provides three cases of plans for a given planning problem P. A plan, $\pi =<a_{1},\ldots ,a_{n}>$, represents a sequence of operator instances of a task operation. The first case of a plan represented by Line 3 denotes an *empty plan* whenever no initial task network is given. Beyond this stage, tasks in the initial task network are chosen nondeterministically, and two different plans are also possible depending on the type of task selected. From Lines 6–17, a primitive task $t_{u}$ that has no predecessors in *t* corresponds to a task, which has an operator instance, and gives the second case of planning. With an assignment of a pair (a,σ), any ground instance *a* of an operator applies to the initial state if only the substitution $name\left(a\right)=\sigma \left(t_{u}\right)$ holds. From Lines 11 and 12, any chosen pair (a,σ) produces a transformed planning problem in which *a* is executed, and the task node of *a* in *t* is removed from the network. In this case, if the plan for the problem P is $\pi =<a_{1},...,a_{n}>$, then the plan for the transformed problem $P^{\prime }$ is $\pi =<a_{2},...,a_{n}>$ when *a* is executed, and $a=a_{1}$.

With the third case of planning, a non-primitive task $t_{u}$ without predecessors in *t* is chosen. On Line 19, a given method instance *m* requires an assignment of the pair (m,σ), such that *m* is applicable to *s* and the substitution name(m)=σ holds. Failure in the assignment automatically terminates the planning process, as Line 21 indicates. Therefore, the plan for $P^{\prime }$ exists if there is a task network $t^{\prime }\in \delta \left(t,u,m,\sigma \right)$ such that the plan is a solution for $\left(s,t^{\prime },O_{pr}\right)$.

### 4.2. Construction of Rules

The model theory-based set semantics of nested concept graphs motivates using a rule-based framework to provide a firm interface between Algorithm 1 and MLN. To that end, logical rules based on FOL translation of OWL statements can provide another view of knowledge constructs that can be used to construct an MLN for MTN task decomposition.

**Figure 5:**
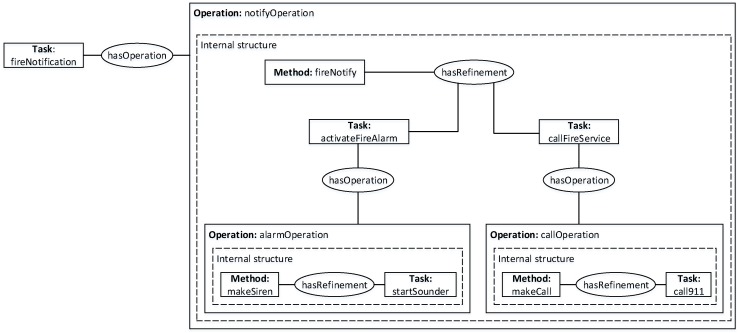
An example of a nested conceptual graph of task decomposition.

Based on Algorithm 1, Figure 5 represents tasks as external information of a concept graph, and a task operation internalizes all applicable decompositions of the task. On this premise, we can build a mapping from nested concept graphs to OWL rules based on the following proposition:

**Proposition** **2.** Regardless of the number of steps of a given task decomposition, the primitive task(s) attained is (are) invariant for a given set of criteria.

**Proof.**  Let *m*, a ground instance of method *M*, use a state decomposition function decompose(x,y) to decompose three given tasks *A*, *B* and *C* as follows:

$$\begin{array}{ccc} A(x)\wedge decompose(x,y) & \Rightarrow & B(y)\\ B(y)\wedge decompose(y,z) & \Rightarrow & C(z) \end{array}$$

When we combine the decompositions of *A* and *B* using the logical and operator, the composite function obtained is:

$$\begin{array}{ccc} decompose(x,y)\wedge decompose(y,z) & \Rightarrow & decompose(x,z) \end{array}$$

Clearly, this is a transitive relation denoting a direct decomposition from *A* to *C*, and hence, the two decompositions involving *A*, *B*, *C* can be represented by:

$$\begin{array}{ccc} A(x)\wedge decompose(x,z) & \Rightarrow & C(z) \end{array}$$

 □

With this mapping, Figure 5 presents each internal structure as a specific task decomposition, and it symbolizes a state function in which primitive tasks depend only on the desired objective instead of the length of decomposition.

### 4.3. MTN Task Decomposition

First, MLN binary predicates are composed into rules based on our framework of concept graphs. These predicates are FOL translations of OWL properties: *hasOperation*; *hasInput*; *hasMethod*; *hasRefinement*; and *requiresOperator*. Thus, these properties form the skeleton of the rules for task decomposition and the assignment of operators to primitive tasks based on our HSS ontology.

For a single-stage task decomposition, the OWL rule in FOL is:

$$\begin{array}{cc} \forall x,y, & Task(x)\wedge hasOperation(x,y)\Rightarrow Operation(y)\\ \forall x,y, & Operation(x)\wedge hasInput(x,y)\Rightarrow Input(y)\\ \forall x,y, & Operation(x)\wedge hasMethod(x,y)\Rightarrow Method(y)\\ \forall x,y, & Method(x)\wedge hasRefinement(x,y)\Rightarrow SubTask(y) \end{array}$$

This rule is a direct manifestation of Proposition 2 and can support both forward and backward chaining of tasks. However, disambiguating between complex and primitive tasks requires amending the above rules as shown below:

$$\begin{array}{cc} \forall x,y, & Task(x)\wedge hasOperation(x,y)\Rightarrow Operation(y)\\ \forall x,y, & Operation(x)\wedge hasInput(x,y)\Rightarrow Input(y)\\ \forall x,y, & Task(x)\wedge requiresOperator(x,y)\Rightarrow Operator(y) \end{array}$$

Obviously, these two rules use method and operator instances to distinguish between primitive and non-primitive tasks.

The second decomposition process extends above rules to achieve recursive decompositions. We provide the following formula as an augmentation:

$$\begin{array}{ccc} decompose(x,y)\wedge decompose(y,z) & \Rightarrow & decompose(x,z) \end{array}$$

For a two-stage decomposition, this augmentation is a direct provision of Proposition 2. The route to addressing recursive decomposition of tasks beyond a two-stage process is a viewpoint of Algorithm 2. As shown on Line 4, proceeding beyond a two-stage decomposition provides that every decomposition stage maps directly to the initial task. For instance, if we consider a sequence of decomposition pairs <(x,y),(y,z),(z,k)>, this algorithm first provides a mapping from *x* to *z* if (x,y)∧(y,z)⇒(x,z) is valid. When this condition is satisfied, the algorithm proceeds by using (x,z) to replace (x,y),(y,z) and forms a new sequence <(x,z),(z,k)>. This new sequence can also be transformed into (x,k), and the process terminates depending on the recursivity of the decomposition.

**Algorithm 2:** Algorithm for recursive decomposition on MTN.** Input:** sequence of task decompositions ** Output:** mapping from initial task to primitive task
 1:**while** successive decompositions exist **do** 2: select first successive decomposition pair: (x,y) and (y,z) 3: **if** (x,y)∧(y,z)⇒(x,z) **then** 4:  replace {(x,y),(y,z)} with (x,z) 5: **end** **if** 6:**end** **while** 7:**return** (x,z)


**Figure 6:**
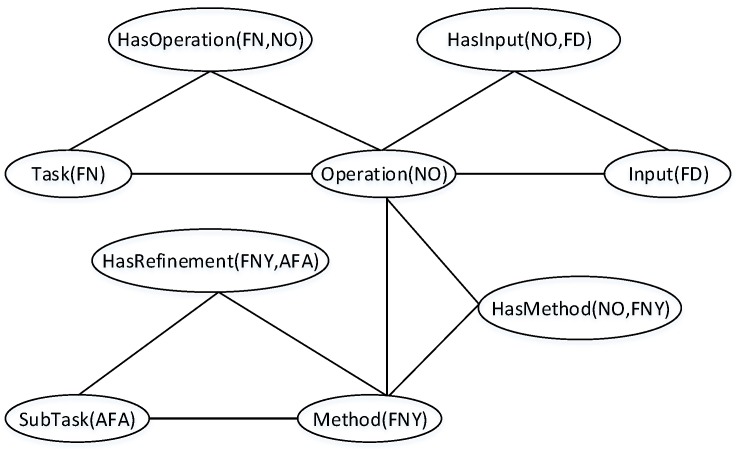
Ground Markov network (MN) obtained by applying MTN for the decomposition of task fireNotification *Task(FN)* into subtask activateFireAlarm *SubTask(AFA)*.

When weights are attached to the predicates of these rules, an MLN is defined, which together with a set of ground atoms defines a ground MN for MTN task decomposition. In Figure 6, an example of a ground MN obtained by applying MTN for task decomposition is given. In this example, the task, fireNotification(FN) is related to notifyOperation(NO) through the property hasOperation. Because these three ground atoms appear together in the same formula in the MLN, they form a clique in the ground MN. This representation gives a triangular maximum clique, which achieves an HTN task decomposition for a given task and a set of criteria whilst addressing both domain complexity and uncertainty. Therefore, reasoning about the subtask *SubTask(AFA)* on this ground MN using Most Probable Explanation (MPE) [16,24] to denote the probability that a decomposition achieves this subtask for a given criteria is given by:

(1) $$\begin{array}{ccc} \arg\max_{SubTask}P\left(SubTask|Task,Input\right) & = & \arg\max_{SubTask}\frac{1}{Z}\exp\left\{\sum_{i}w_{i}n_{i}\left(Task,Input,SubTask\right)\right\}\\ & = & \arg\max_{SubTask}\sum_{i}w_{i}n_{i}\left(Task,Input,SubTask\right) \end{array}$$

where $n_{i}\left(Task,Input,SubTask\right)$ is the number of true grounding of all formulae, $w_{i}$ is the weight of each formula and *Z* is the normalization constant. This can also be used to answer conditional probability queries that the formula containing the subtask *SubTask(AFA)* holds for a given evidence *Task(FN)* and *Input(FD)* as follows:

(2) $$\begin{array}{ccc} P(SubTask(x)|Task(x),Input(x),C) & = & P(SubTask\left(AFA\right)|Task\left(FN\right),Input\left(FD\right),M_{LC})\\ & = & \frac{P(SubTask\left(AFA\right)\wedge Task\left(FN\right)\wedge Input\left(FD\right)|M_{LC})}{P(Task\left(FN\right)\wedge Input\left(FD\right)|M_{LC})}\\ & = & \frac{\sum_{\omega \in \Omega_{SubTask}\cap \Omega_{Task}\cap \Omega_{Input}}P\left(\alpha =\omega |M_{LC}\right)}{\sum_{\omega \in \Omega_{Task}\cap \Omega_{Input}}P\left(\alpha =\omega |M_{LC}\right)} \end{array}$$

where $\Omega_{SubTask}$, $\Omega_{Task}$ and $\Omega_{Input}$ denote that the worlds the three formulae hold, respectively, *C* denotes the set of constants, $M_{LC}$ is the ground Markov network defined by the formulae and the set of constants and $P(\alpha =\omega |M_{LC})$ is computed as:

(3) $$P\left(\alpha =\omega \right)=\frac{1}{Z}\exp\left\{\sum_{i}w_{i}n_{i}\left(\alpha \right)\right\}=\frac{1}{Z}\prod_{i}\phi_{i}{\left(\alpha_{\left\{i\right\}}\right)}^{n_{i}\left(\alpha \right)}$$

This is similar to conditional probabilities in graphical models, and all predicates in the formulae and the MLN are zero-arity.

## 5. Experimental Design

We provide details of our experimental design in this section. A use case of automatic fire control in a smart home is presented, and an algorithm for the generation of datasets for our experiments is discussed.

### 5.1. Use Case

Increasingly, the need for reliable automatic fire control systems inspires the use of automated HTN planning in smart homes. With the myriad of interconnected devices and agents, these systems generally function in domains that are inherently complex and uncertain. Even though the adoption of ontological modeling as a semantic technology can efficiently address domain complexity to achieve interoperability, a major difficulty is that ontology in its classical form does not support uncertainty reasoning. Therefore, leveraging CPSs that automatically compose services to solve complex tasks must be designed and operated under uncertainty to increase robustness and adaptability to context.

HTN planning under uncertainty provides a novel extension of deterministic planning in which a smart home’s activities can be monitored, planned and executed non-deterministically. The main difference with traditional HTN planning is that the decomposition of tasks, such as fireNotification, represents a ground MN of rules rather than a detailed sequence of subtasks. Intuitively, this network softens the classical decomposition criteria of HTN and represents how likely it is that a given task results into a subtask for a given criterion.

**Figure 7:**
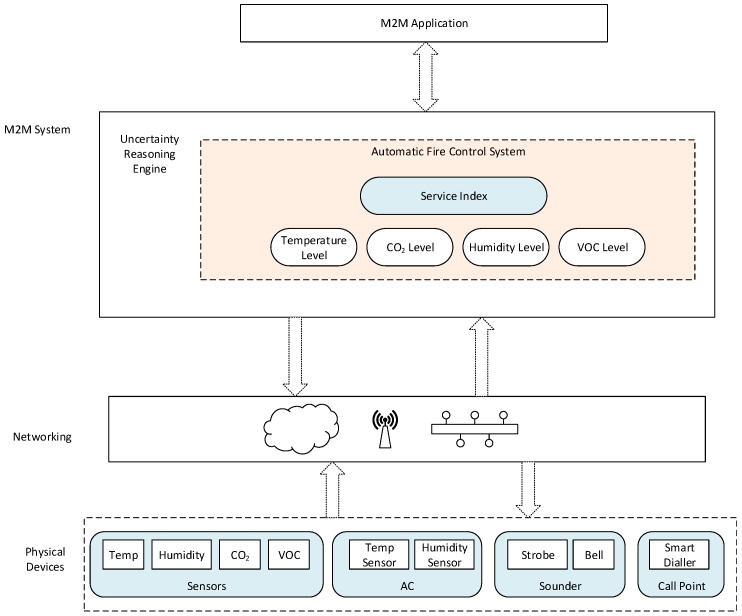
Use case of an automatic fire control system.

As shown in Figure 7, a use case of an automatic fire control application is described. Through ontological modeling, this system has knowledge about all structural elements and equipment installed in the home. This means devices in the home can easily be discovered using their deployment information, and safety conditions related to automatic fire control can be effectively monitored in the home.

As can be seen in this use case, distributed sensor networks underpin the semantic capabilities of this design, and through semantic reasoning, high-level system goals can be inferred from low-level contextual information. The high-level goals automatically generate a plan of services that achieve such goals by issuing commands to controllers. These services can represent complex tasks that, in order to be executed, are recursively decomposed on-the-fly into commands that control processes. As a CPS, this is an integrated process, which works in a closed loop with underlying semantic agents that directly control the home’s devices, e.g., the actuators that dial emergency services’ numbers and those that control fire alarms.

Given the criticality of preventing fire disasters in smart homes, it is important to understand the phenomenon of uncertainty in this use case. To this end, sensitivity to timing and the nature of inputs are two sources of uncertainty that can affect the reliability of these systems. For instance, an agent detecting smoke from a nearby kitchen, coupled with a high temperature at its deployment, can trigger a false fire alarm. This could be disastrous when a false negative is encountered, and leveraging MTN in this regard can address both domain uncertainty and complexity towards efficient system calibration with desired precision. Thus, automated planning under uncertainty can essentially make us realize the full benefits of this innovative technology towards dynamic service composition in CPSs.

Service composition on this use case requires that contextual information is reasoned upon to attain high-level system goals for further processing in the reasoning engine. Owing to the heterogeneity of the data acquired from the environment, semantic modeling of non-actuator devices is adopted to describe those devices in terms of their measurement capability, measured value and location in a context ontology. This ontology models a *Device* class as a thing type and key properties include *hasDeployment*, *hasCapability* and *hasValue* to respectively specify the environment, capability and output perspectives of devices. Low-level contextual information after semantic annotation is aggregated with the context ontology to form a coherent model that leverages domain knowledge to infer implicit knowledge. This implicit knowledge denotes the high-level system goals, which are central in initiating fire control activities in the use case through service composition based on MTN. These services can specify the real-time demand responsiveness of systems’ components in terms of interfaces and observations [25], and it is therefore important composing services of predefined operations, which are desirable to both systems and users of CPSs.

The basic idea serving as a point of departure in MTN on this use case lies in the fact that logic rules for composing MLN can be generated by semantically modeling the use case based on the HSS ontology. Properties and concepts of this ontology provide relations between tasks and their decomposition criteria and define binary MLN predicates, which constitute the task decomposition rules of MTN. Whilst a primitive task represents an operator instance in the HSS ontology, complex tasks can be refined into primitive tasks using these rules. For example, the logic rules for decomposing a high-level task fireNotification into its subtasks are shown below:

$$\begin{array}{c} Task(fireNotification)\wedge hasOperation(fireNotification,notifyOperation)\Rightarrow Operation(notifyOperation)\\ Operation(notifyOperation)\wedge hasInput(notifyOperation,powerSupply)\Rightarrow Input(powerSupply)\\ Operation(notifyOperation)\wedge hasMethod(notifyOperation,fireNotify)\Rightarrow Method(fireNotify)\\ Method(fireNotify)\wedge hasRefinement(fireNotify,activateFireAlarm)\Rightarrow Task(activateFireAlarm) \end{array}$$

With associated weights; these are ground MLN predicates of concepts and properties of the HSS ontology, and each rule defines the relation between concepts appearing together in the same MLN formula. As we can see, these rules specify task decomposition constraints using the input property that requires a constant power supply before a subtask for fire alarm activation can be achieved. The weighted ground rules define a ground MN, and reasoning about the subtask on the MN denotes the probability that the MLN achieves this subtask for the given rules. This probability is a measure of the precision of service composition under uncertainty, and proper calibration of systems is an inherent property of the training dataset of the MLN.

### 5.2. Task Generation

By formulating task decomposition rules based on MTN, training and test datasets are required to validate the model’s performance. As shown in Algorithm 3, synthetic datasets are possible in the absence of real data to meet the requirements of the underlying ontology of MTN. All datasets generated contain the decomposition structure and constraints of tasks. Note that we avoid human biases in the generation process by employing a randomized task generation approach in which the number of subtasks at each level of decomposition is not predefined.

**Algorithm 3:** Algorithm for task generation for MTN.**Input:**
taskNumber, recursion **Output:** Decomposition structure and constraints of tasks
 1:**while** taskNumber≠∅ **do** 2: Task← generate: Task(taskNumber) 3: **if** recursion>1 **then** 4:  recursion←Random(1,recursion) 5: **end** **if** 6: **while** recursion>0 **do** 7:  generate: Operation(Task(taskNumber)) 8:  generate: Input(Task(taskNumber)) 9:  generate: Method(Task(taskNumber)) 10:  generate: SubTask(Task(taskNumber)) 11:  Task←SubTask(Task(taskNumber)) 12:  recursion←recursion-1 13: **end** **while** 14: taskNumber←taskNumber-1 15:**end** **while** 16:**return** task and constraints


The initial task network and maximum allowed number of decompositions at each stage are inputs to the algorithm. When the initial task network is specified, generate a task and proceed to determine the number of associated subtasks (Line 2). For each task, the number of associated subtasks is a random number generated between one and the maximum allowed number of decompositions (Line 4). This is a strategy adopted to avoid human biases in the number of decompositions for a given task. Until the maximum number of decompositions, generate constraints and subtasks (Lines 6–13). A special case in this regard involves a maximum allowed decompositions of one, and it is the case of a single-stage decomposition. This requires the algorithm to terminate after generating the first subtasks and their constraints for every task. Datasets for recursive decomposition are also generated when the number of decompositions exceeds one, and the length of decompositions are arbitrary for tasks in the network. In both cases, independent training and test datasets can be generated based on this algorithm.

## 6. Results and Discussion

This section discusses the results of experiments performed to evaluate the feasibility of our approach. Task decomposition rules were formulated, and different synthetic datasets were generated to train and test the performance of the underlying MLN towards evaluating the feasibility of task decomposition on MTN. These datasets varied in the number of ground terms and described tasks and their methods for single-stage and recursive decompositions. Note that the training and test datasets were generated independent of each other without any fixed relationship in their patterns. In dealing with uncertainty in the datasets, we assumed a lack of certainty about the timing and nature of contexts from which high-level system goals are elicited. As such, these goals are also uncertain, and knowledge about future outcomes based on the same becomes unpredictable.

**Figure 8:**
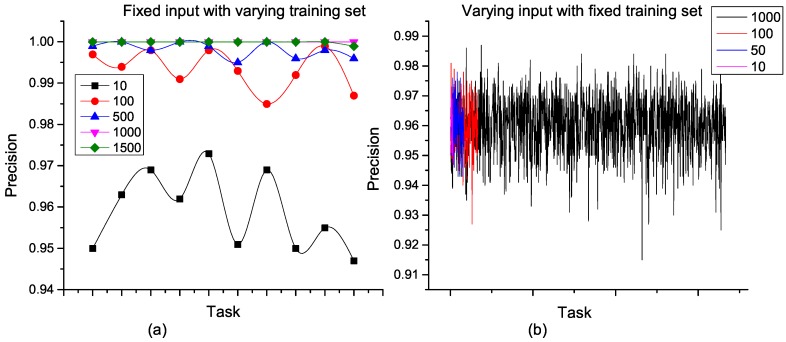
Single-stage task decomposition with single-stage training sets. (**a**) Fixed input with varying training set; (**b**) Varying input with fixed training set.

In the experiments, weights of formulae in the underlying MLN were learned using the training datasets and the accuracy of decomposition validated using the test datasets. To measure the accuracy of each decomposition strategy, we used the marginal probabilities of subtasks given high-level tasks as evidence. As shown in Figure 8, the probability that a subtask is correct after MTN single-stage decomposition is presented. This probability represents the precision of decomposition, and values for different single-stage training and test sets were measured. From Figure 8a, the precision exceeds 94% for a fixed input task irrespective of the number of constants in the MLN training set. Typical of MLN, the performance of the MTN-based single-stage task decomposition improves as more constants are considered in the training set. In this case, optimal performance was obtained for training sets with at least 1000 constants. However, when the training set is fixed for a varying number of input tasks, the performance is steady. We see in Figure 8b that varying the number of input tasks between 10 and 1000 in the training set gave an average precision above 95% in all cases. This clearly shows that task decomposition based on MTN scales very well with the amount of input task with the best performance.

**Figure 9:**
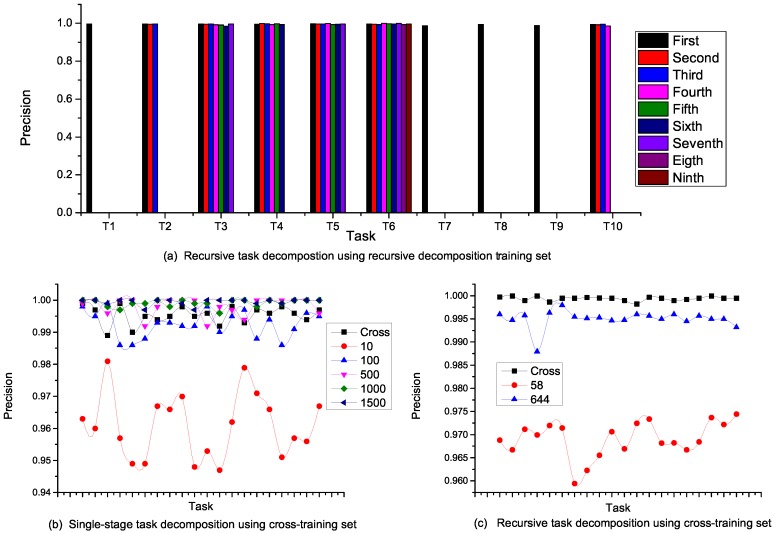
Recursive decomposition and cross-validation of single-stage decomposition and recursive decomposition. (**a**) Recursive task decompostion using recursive decomposition training set; (**b**) Single-stage task decomposition using cross-training set; (**c**) Recursive task decomposition using cross-training set.

Because a task operation internalizes all decompositions of a given task, the precision is an internal structure, which is fairly constant for all stages of recursive decomposition. As shown in Figure 9a, the propagation of precision in a recursive decomposition of a 10-task input is presented. Each task was constrained to a maximum of ten subtasks, and we measured the precision of all decompositions between the initial and final tasks. Tasks T1, T7, T8 and T9 attained primitive tasks after the first stage of decomposition. For T6, this went through nine successive decompositions, and the precision at each stage was fairly constant. This observation cuts across all tasks with recursive decompositions, and a precision of 99% was achieved at all levels of decomposition.

Each nesting of a recursive decomposition represents a single-stage decomposition and provides a single view of multiple task inputs in a recursive decomposition as a unit with an approximately high constant precision value. In this view, the performance of this approach does not depend on the category of training set considered. As we can see in Figure 9b,c, when a recursive decomposition training set is adopted for a single-stage decomposition and vice versa, the performance only depends on the number of ground terms considered. In the cross-training recursive decomposition for instance, the set *Cross* contained 3000 constants and expectedly outperformed the other sets with a lesser number of constants.

**Figure 10:**
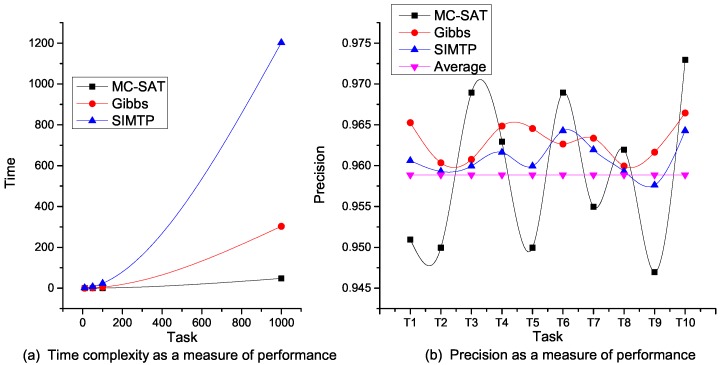
Performance of propositional and lazy probabilistic inference algorithms. (**a**) Time complexity as a measure of performance; (**b**) Precision as a measure of performance.

Further, we evaluated the performance of task decomposition on MTN using three MLN propositional and lazy probabilistic inference algorithms [16,24]. As shown in Figure 10, whilst it takes the algorithm which combines Markov chain Monte Carlo and satisfiability (MC-SAT) about 1 s to decompose up to 1000 input tasks, both Gibbs sampling and simulated tempering (SIMTP) require over 5 min to achieve the same results. However, the frequency spectrum of the precision involving Gibbs sampling and SIMTP is smoother than that of MC-SAT. Clearly, these two algorithms represent a good fit for the average precision of MC-SAT, and thus, this statistically makes MC-SAT suitable for CPSs where demand responsiveness is key.

From the above analyses, it is clear that different task decomposition strategies are possible with our approach. The overall precision of above 90% for decomposing large task inputs in seconds manifests the feasibility in reducing both computational and inferential load in service composition under uncertainty for CPSs. Overall, this approach is ideal for automated reasoning techniques that can dynamically interpret workflow to achieve demand responsiveness in CPSs.

## 7. Related Work

Using computation, communication and control to expand capabilities that can interact with physical objects drives the advancement of CPSs. Research challenges include the design and development of uncertainty-based interoperable models that can represent both the cyber and physical components with high fidelity. However, the growing complexity of CPSs often restricts most approaches to component-based modeling [26,27]. Typically, it becomes very challenging to assess component-to-component physical and behavioral interactions at the system level when component-based modeling is adopted [28]. Salient techniques that address the requirements of both the cyber and physical components using SOA include [4]. Using only the SOA, however, is not ideal for modeling real-time distributed CPSs, whilst agent-based techniques are possible [6].

Because of the complex dynamics of CPSs, the use of semantics and distributed agents in CPSs motivates this approach. A typical semantic multi-agent architecture for CPSs is used to model both the structure and behavior of an intelligent water distribution system in [29]. This model, however, provides no mechanism for task allocation and dynamic service composition in CPSs. However, the problem of composing services to achieve complex system tasks has become practical in CPSs [10] and other important enterprise integration, such as business-to-business processes [11,12]. In addition, partial observation and limited domain knowledge are practical in CPSs, and using certain-based techniques to control these uncertain environments will not lead to the maximum benefit of CPSs [13,14].

In our approach, therefore, we adopt an artificial intelligence planning technique to dynamically form collaborative services in CPSs. In dealing with the inherent domain complexity and uncertainty, we further propose a technique that leverages the inferential power of MLN into the dynamic task decomposition of HTN. This novel integration can reduce both computational and inferential loads in service composition in CPSs.

## 8. Conclusions

In this paper, we proposed a cross-layer automation and management model towards the dynamic composition of services in CPSs. Using ontological intelligence, we further proposed a novel bridge between HTN and MLN to achieve a dynamic service composition under uncertainty. Essentially, this bridge leverages the efficient inference algorithms of MLN to define an HTN task decomposition strategy that can achieve reliable functioning of CPSs in complex and uncertain physical domains. From the results of our experiments, this approach can reduce both inferential and computational loads in automated planning towards the efficient composition of services in CPSs. We recognize that since all of our results were obtained from synthetic data, future work of our research shall consider a case study of a real domain.

## Figures and Tables

**Listing 1:**
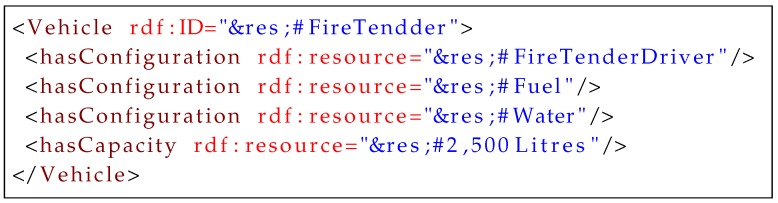
Example of resource modeling.

**Listing 2:**
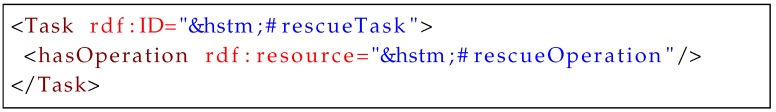
Example of task modeling.

**Listing 3:**
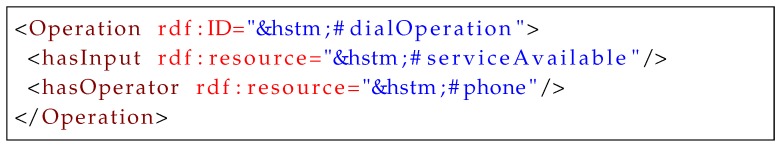
Task operation of an operator.

**Listing 4:**
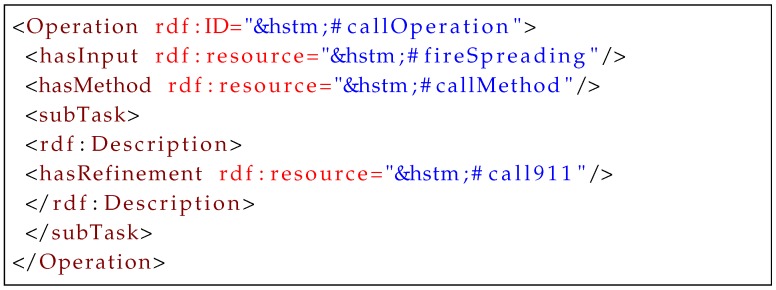
Task operation of a method.

**Table 1:** Translation of OWL concepts into first-order logic (FOL).

Concept	OWL Syntax	FOL
Class	owl:Class	C(x)
Special class	owl:Thing	x=x
Empty class	owl:Nothing	¬(x=x)
Intersection of concepts	owl:intersectionOf	$C_{1}\left(x\right)\wedge C_{2}\left(x\right)$
Union of concepts	owl:unionOf	$C_{1}\left(x\right)\vee C_{2}\left(x\right)$
Complement of concepts	owl:complementOf	¬C(x)
Property	rdf:Property	P(x,y)⇒C(x)∧C(y)
Universal restriction	owl:allValuesFrom	∀y,P(x,y)⇒C(y)
Existential restriction	owl:someValuesFrom	∀y,P(x,y)∧C(y)

**Table 2:** Translation of OWL axioms to FOL.

Axiom	OWL Syntax	FOL
type	rdf:type	$C(X_{1})$
subClassOf	rdfs:subClassOf	$\forall x,C_{1}\left(x\right)\Rightarrow C_{2}\left(x\right)$
sameClassAs	owl:sameClassAs	$\forall x,C_{1}\left(x\right)\Rightarrow C_{2}\left(x\right)$
		$\forall x,C_{2}\left(X\right)\Rightarrow C_{1}\left(X\right)$
subPropertyOf	rdfs:subPropertyOf	$\forall x,y,P_{1}\left(x,y\right)\Rightarrow P_{2}\left(x,y\right)$
Property domain	rdfs:domain	∀x,y,P(x,y)⇒C(x)
Property range	rdfs:range	∀x,y,P(x,y)⇒C(y)
inverseOf	owl:inverseOf	$\forall x,y,P\left(x,y\right)\Rightarrow P^{1}\left(y,x\right)$
		$\forall x,y,P^{1}\left(y,x\right)\Rightarrow P\left(x,y\right)$
SymmetricProperty	owl:SymmetricProperty	∀x,y,P(x,y)⇒P(y,x)
		∀x,y,P(y,x)⇒P(x,y)
FunctionalProperty	owl:FunctionalProperty	∀x,y,zP(x,y)⇒P(x,z)
		∀x,y,zP(x,z)⇒P(x,y)
InverseFunctionalProperty	owl:InverseFunctionalProperty	$\forall x,y,zP\left(x,y\right)\Rightarrow P^{1}\left(z,y\right)$
		$\forall x,y,zP^{1}\left(z,y\right)\Rightarrow P\left(x,y\right)$
TransitiveProperty	owl:TransitiveProperty	∀x,y,z,P(x,y)∧P(y,z)⇒P(x,z)
